# ﻿*Acronema handeloides*, a new species from Jiaozi Mountain, Yunnan Province, China (Apiaceae)

**DOI:** 10.3897/phytokeys.267.174758

**Published:** 2025-12-02

**Authors:** Xu-Dong Ma, Hui-Min Li, Ying Xie, Jun-Wen Zhu, Jun Wen, Wei Zhou, Bao-Cheng Wu, Chun-Feng Song

**Affiliations:** 1 Jiangsu Key Laboratory for Conservation and Utilization of Plant Resources, Institute of Botany, Jiangsu Province and Chinese Academy of Sciences (Nanjing Botanical Garden Mem. Sun Yat-Sen), Nanjing 210014, China Institute of Botany, Jiangsu Province and Chinese Academy of Sciences Nanjing China; 2 CAS Key Laboratory for Plant Diversity and Biogeography of East Asia, Kunming Institute of Botany, Chinese Academy of Sciences, Kunming 650201, China Kunming Institute of Botany, Chinese Academy of Sciences Kunming China

**Keywords:** *
Acronema
*, alpine plants, new species, taxonomy, Yunnan

## Abstract

*Acronema handeloides*, a new species of Apiaceae from Jiaozi Mountain in Yunnan Province, is described and illustrated herein. This species is similar to *A. handelii* and *A. sichuanense* but can be distinguished by its 1–2 branches; variable basal leaves; bracts 1 or occasionally present, bracteoles 1–3; subulate calyx teeth; linear-subulate, papillate petal apex; and broadly ovoid. Phylogenetic analyses also support that it is a monophyletic group sister to *A. handelii* and *A. sichuanense*. In accordance with the IUCN Red List criteria (2024), the conservation status of *A. handeloides* is preliminarily assessed as Data Deficient (DD).

## ﻿Introduction

*Acronema* Edgew. is distributed from the southern Himalayan region to the Hengduan Mountains. According to the Flora of China and subsequent records of new species and discoveries in recent years (Pan et al. 2005; Wang et al. 2013), approximately 22 species are currently recognized in China. The genus is characterized by its petals, which possess a filamentous and straight apex. Most species of *Acronema* are typically found in pristine alpine forests above 3,000 meters, making them difficult to encounter in early surveys. Due to the scarcity of specimens and the generally poor quality of available collections, this genus has always remained insufficiently studied within the Apiaceae.

Due to the complex topography of the Hengduan Mountains, where high peaks and deep valleys form unique “Sky Island” ecosystems, the region exerts a profound influence on plant diversification and genetic structure (Zhang et al. 2021; Rana et al. 2025). Like many other alpine understory species, the genus *Acronema* exhibits a fragmented distribution across different mountain ranges and has gradually diversified into several unique species. It is common to find multiple *Acronema* species distributed at different altitudes on the same mountain. In 2024, during a survey of *A. crassifolium* Huan C.Wang, X.M.Zhou & Y.H.Wang on Jiaozi Mountain (Luquan County, Kunming, Yunnan), we discovered an extremely small plant initially suspected to be *A. handelii* H.Wolff or *A. sichuanense* S.L.Liou & R.H.Shan, characterized by a globose root and broadly ovate fruits with calyx teeth. To clarify its taxonomic status, we conducted further fieldwork in 2025, focusing on the morphology of petals and basal leaves. Our observations confirmed that this species is distinct from all known *Acronema* species.

Since Zhou Jing inferred the phylogeny of the subfamily Apioideae (Apiaceae) using ITS sequences and the rps16 and rpl16 intron sequence variation, the phylogenetic position of the genus *Acronema* received clarification within this subfamily (Zhou et al. 2008, 2009). Subsequently, Chen Lian employed plastome data and ITS sequences to further resolve interspecific relationships among 16 *Acronema* species (Chen et al. 2024). To clarify the phylogenetic position of this species, we performed phylogenetic analyses based on ITS sequence data from 7 *Acronema* species, and the results confirmed our hypothesis. Due to its overall similarity in floral and fruit characteristics to *A. handelii*, we describe it herein as a new species, *A. handeloides* X.D.Ma & C.F.Song.

## ﻿Material and methods

### ﻿Species sampling

In late August 2024, fruiting specimens were collected from Jiaozi Mountain in Luquan County, Kunming, Yunnan. Fresh ripe fruits were photographed in situ using an Olympus TG6 camera, and the number of oil ducts was examined. In late July 2025, flowering individuals were revisited at the same site, with emphasis on collecting specimens representing morphological variation within the population. Fresh petal characteristics were documented with the same camera. Subsequent morphological measurements and counts were conducted with ImageJ to supplement the specimen records.

### ﻿DNA sequencing

We assembled the nuclear ribosomal internal transcribed spacer (nrITS) of *A. handeloides* to determine its phylogenetic position within the genus. In total, 7 currently recognized species of *Acronema* and 4 currently recognized species of the *Acronema* clade (*Sinocarum cruciatum* (Franch.) H.Wolff, *S. filicinum* H.Wolff, *S. muliense* L.J.Gui, Y.P.Xiao & X.J.He, *S. vaginatum* H.Wolff) were included, with two additional species (*Meeboldia delavayi* (Franch.) W.Gou & X.J.He, *M. yunnanensis* (H.Wolff) Constance & F.T.Pu ex S.L.Liou) selected as outgroups. Sequences of *A. handeloides*, *A. chinense*, *A. minus*, *A. sichuanense*, and *A. handelii* were newly generated in this study, while the remaining sequences were retrieved from GenBank. Detailed information on the material sources and GenBank accession numbers is provided in Tables 1, 2.

**Table 1.** Newly generated sequences.

Taxon	Locality	Voucher
*Acronema handeloides*	China, Yunnan, Kunming	MXD370-1 (NAS)
*Acronema handeloides*	China, Yunnan, Kunming	MXD370-4 (NAS)
*Acronema handelii*	China, Yunnan, Diqing	MXD364-2 (NAS)
*Acronema handelii*	China, Yunnan, Diqing	MXD364-15 (NAS)
*Acronema sichuanense*	China, Sichuan, Ngaba	MXD365-2 (NAS)
*Acronema sichuanense*	China, Sichuan, Ngaba	MXD365-9 (NAS)
*Acronema sichuanense*	China, Sichuan, Ngaba	MXD365-12 (NAS)
*Acronema chinense*	China, Sichuan, Ngaba	MXD366-1 (NAS)
*Acronema chinense*	China, Sichuan, Ngaba	MXD366-6 (NAS)
*Acronema minus*	China, Yunnan, Kunming	MXD290a-1 (NAS)
*Acronema minus*	China, Yunnan, Kunming	MXD372-1 (NAS)
*Acronema minus*	China, Yunnan, Kunming	MXD372-2 (NAS)

**Table 2.** Source of materials studied and GenBank accession numbers.

Taxon	Voucher	ITS
*Acronema muscicola*	XZ2011081741 (SZ)	KP940756
*Acronema schneideri*	YLDP244A	MH117405
*Acronema graminifolium*	1	MT359945
*Meeboldia delavayi*	G19072401 (SZ)	MN688992
*Meeboldia yunnanensis*	G18071908 (SZ)	MN688997
*Sinocarum cruciatum*	XYP19080301	MN846686
*Sinocarum vaginatum*	XYP19080402	MN846688
*Sinocarum muliense*	GLJ18081101_1	MZ054145
*Sinocarum muliense*	GLJ18081101_2	MZ054146
*Sinocarum filicinum*	XYP19080302	MT586806
*Sinocarum filicinum*	XYP19091803	MT586807

The total genomic DNA of *A. handeloides* was extracted by Wuhan Benagen. The nrITS region was assembled using GetOrganelle v.1.7.7.1 (Jin et al. 2020). Geneious Prime 2024.0.2 (Kearse et al. 2012) was used to annotate the assembled plastomes and extract the ITS1, 5.8S, and ITS2 regions used in the phylogenetic analysis. The MAFFT alignment plug-in of Geneious was used to align the 23 sequences obtained, using the default parameters.

IQ-TREE 2 (Bui et al. 2020) was used, and the K2P+G4 model was selected to construct a maximum likelihood (ML) tree with 1000 bootstrap replicates. In the ML analysis, bootstrap support (BS) values < 50% were considered unreliable, values between 50% and 75% were considered moderately supported, and values > 75% were considered strongly supported. BI analyses were conducted using MrBayes v.3.2.7 (Ronquist et al. 2012) under the selected JC model with the following settings: mcmc ngen = 2,000,000; nruns = 2; nchains = 4; samplefreq = 1000; temp = 0.2; sumt burnin = 2000. In the BI analysis, posterior probability (PP) values < 0.90 were considered poorly supported, values between 0.90 and 0.95 were considered moderately supported, and values > 0.95 were considered strongly supported. The final sequence alignments have been deposited in the Zenodo repository (DOI: https://doi.org/10.5281/zenodo.17451735).

## ﻿Results and discussion

### ﻿General morphology

*Acronema handeloides*, *A. handelii*, and *A. sichuanense* are all short herbs with globose roots, petals featuring filiform, papillate tips, prominent calyx teeth, and broadly ovate fruits. However, *A. handeloides* exhibits less developed leaf forms, ranging from simple to trifoliate or ternate-bipinnate. In contrast, the basal leaves of *A. handelii* are mono- to bipinnate, while those of *A. sichuanense* are more consistently ternate-bipinnate. Beyond leaf morphology, the three species also differ in the number of umbels and umbellules. *Acronema handeloides* has fewer of both, typically bearing 2–4 (rarely up to 5) umbels and umbellules. *Acronema handelii*, on the other hand, possesses 4–6 (often 5) umbels and 3–9 (often 6) umbellules, and *A. sichuanense* has 3–6 (often 5) umbels and 3–10 (often 8) umbellules. Regarding petal morphology, all three species possess papillae at their petal apex. However, *A. handeloides* more closely resembles *A. handelii* in having ovate-lanceolate petals, which clearly distinguish them from the ovate petals of *A. sichuanense*. The most critical distinguishing feature is that *A. handeloides* possesses 1–2 branches and 1–3 bracteoles. Within the genus, these characteristics are shared only with *A. tenerum* (DC.) Edgew. and *A. minus* (M.F.Watson) M.F.Watson & Z.H.Pan, while both *A. handelii* and *A. sichuanense* lack these features.

### ﻿Phylogenetic analyses

**Figure 1.**
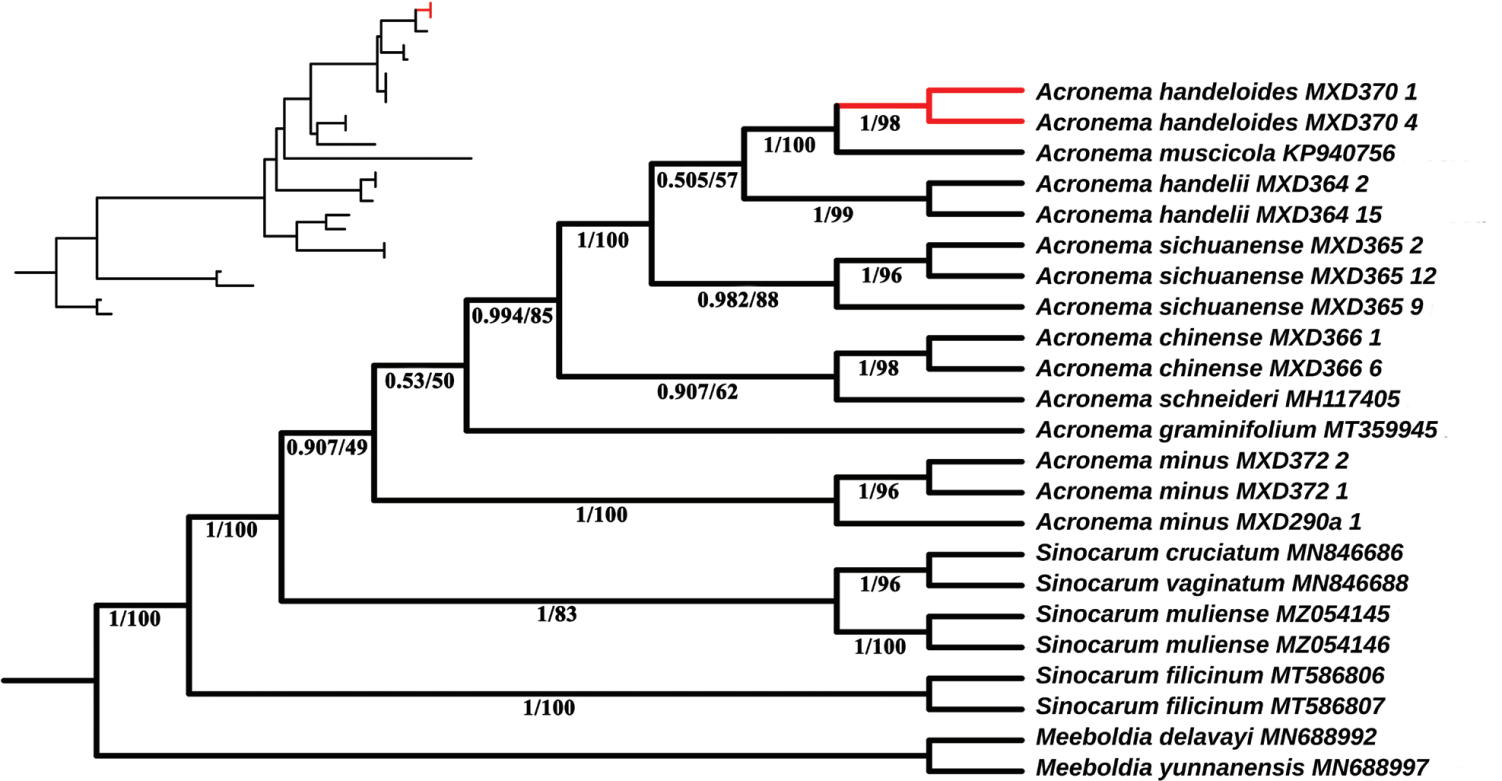
Phylogenetic tree inferred from the ITS sequences. Bayesian posterior probability values (PP) / bootstrap support values (BS) are shown below the branches.

In the phylogenetic analysis, species in the genus *Acronema* formed a monophyletic group, clearly distinguishable from those in the genus *Sinocarum*, and exhibited strong support (PP = 1, BS = 100%). Within *Acronema*, *A. handeloides*, *A. handelii*, and *A. sichuanense* each clustered into three independent monophyletic clades. The high support of each clade indicates that all three species are monophyletic groups with clear genetic differentiation boundaries, strongly supporting the taxonomic status of *A. handeloides* as a valid species. Furthermore, these three morphologically similar species formed a monophyletic group that clustered into the same clade, exhibiting the highest support (PP = 1, BS = 100%), further confirming their close phylogenetic relationship (Fig. 1). Notably, *A. muscicola* (KP940756) was placed within this clade, but its true phylogenetic position could not be determined based on a single specimen. This uncertainty is exacerbated by the significant inconsistency between its known morphological features (e.g., trifoliate basal leaves, thick papery leaf blades, and completely sheathed petioles) and those of all other taxa within this clade. Phylogenetic evidence from Chen Lian (Chen et al. 2024) (a phylogenetic tree based on ITS sequences) further confirms that the phylogenetic relationships of *A. muscicola* require further molecular verification. After excluding the highly uncertain *A. muscicola*, the cluster of *A. handeloides*, *A. handelii*, and *A. sichuanense* exhibits a branch length < 0.01 coupled with low node support. This combined pattern, characterized by extremely short branches and insufficient node support, represents a classic molecular signature of recent rapid radiative evolution. In this scenario, incomplete lineage sorting leads to conflicting phylogenetic signals captured by different phylogenetic methods, a typical feature of recently divergent groups. Therefore, further research is needed to determine the exact sister-group relationships of *A. handeloides*. Specifically, multi-gene joint analysis of nuclear and chloroplast genes in *Acronema* and coalescent simulation analysis should be employed, thereby enabling the definitive identification of the sister group of *A. handeloides*.

### ﻿Taxonomic treatment

#### ﻿ Acronema handeloides

Taxon classificationPlantaeApialesApiaceae

X.D.Ma & C.F.Song sp. nov.

9B983591-5DC5-55ED-A9FD-FBA449E20B63

urn:lsid:ipni.org:names:77370065-1

Figs 3, 4

##### Diagnosis.

**Figure 2.**
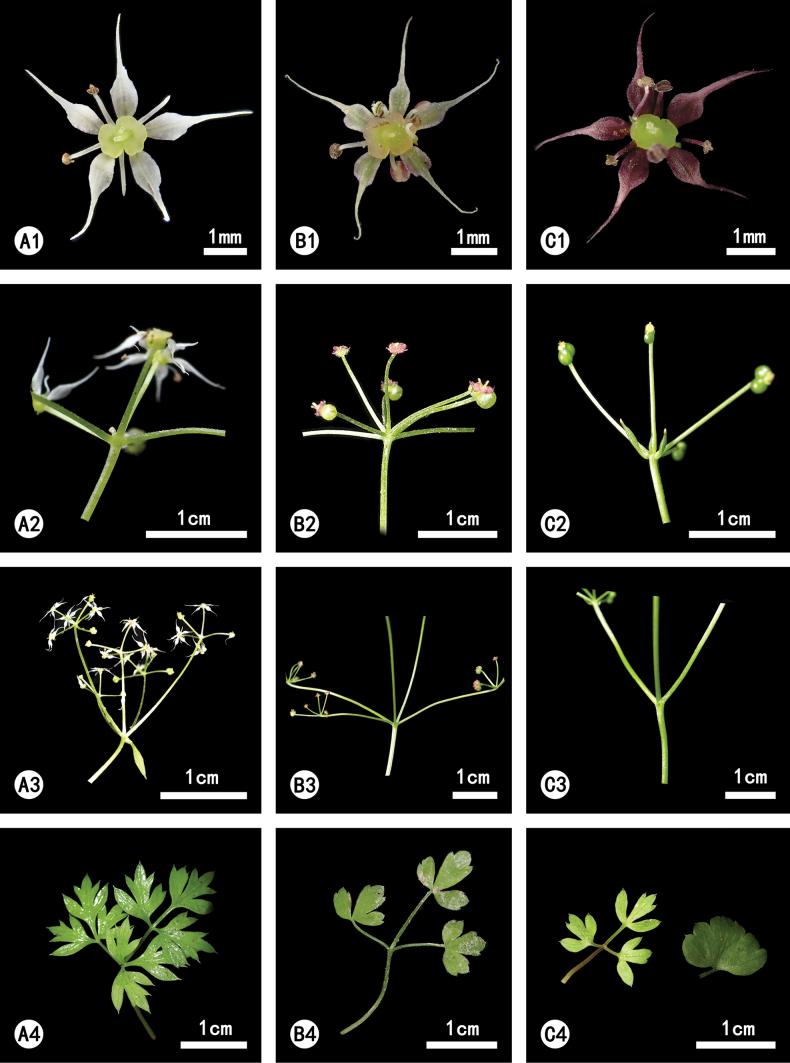
Morphological comparisons between *Acronema handelii* (**A**), *A. sichuanense* (**B**), *A. handeloides* (**C**). **A1–C1.** Flower; **A2–C2.** Bracteoles and umbellules; **A3–C3.** Bracts and rays; **A4–C4.** Basal leaves. Photograph credits: X.D. Ma.

*Acronema handeloides* has flowers and fruits similar to *A. handelii* and *A. sichuanense* but differs from these latter in its frequently branched stems (1–2 branches), fewer number of umbel rays and umbellule rays, 1–3 bracteoles, and variable shape of basal leaves. These three species are readily distinguishable by key morphological characteristics, as detailed in Table 3 and Fig. 2.

**Table 3.** Comparison of morphological characters between *Acronema handeloides*, *A. handelii*, and *A. sichuanense*.

Characters	*Acronema handelii*	*A. sichuanense*	*A. handeloides*
Basal leaves	mono- to 2-ternate pinnate	2-ternate-pinnate	Simple leaf to trifoliolate to 2-ternate pinnately compound leaf
Plant branching	Stem solitary	Stem solitary	Often with 1–2 branches
Number of umbel rays and umbellule rays	Umbels with 4–6 rays (often 5), umbellules with 3–9 rays (often 6)	Umbels with 3–6 rays (often 5), umbellules with 3–10 rays (often 8)	Umbels with 2–4 rays (often 3, rarely up to 5), umbellules with 2–5 rays (often 3)
Bracts and bracteoles	Bracts and bracteoles absent	Bracts and bracteoles absent	Bracts 1 or occasionally present, bracteoles 1–3
Petal characteristics	Ovate-lanceolate, apex linear-subulate, papillate	Obovate, apex linear-subulate, papillate	Ovate-lanceolate, apex linear-subulate, papillate

**Figure 3.**
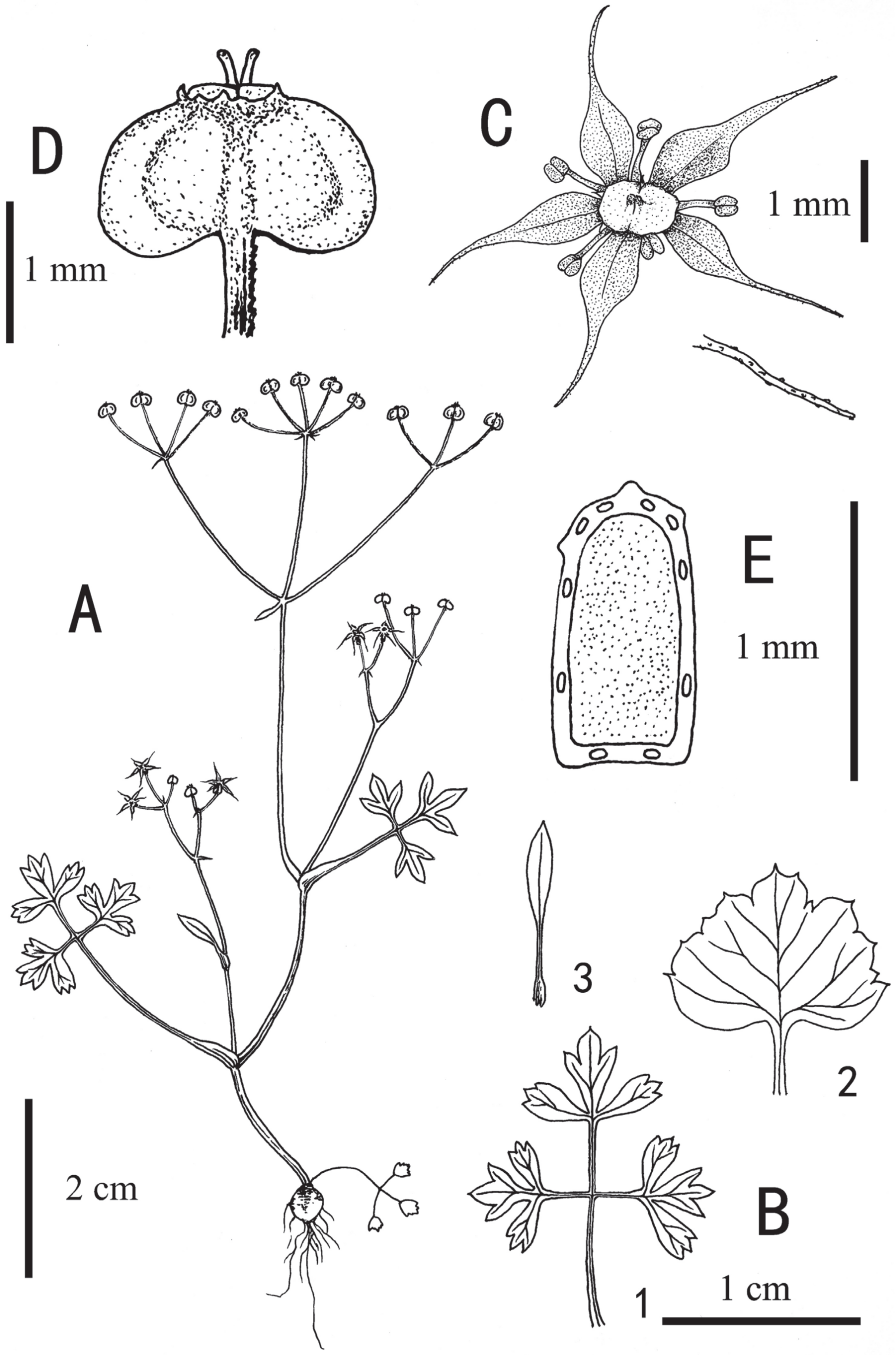
*Acronema handeloides* X.D.Ma & C.F.Song. **A.** Habit; **B.** Basal leaves and stem leaves (**B1, B2, B3** show different leaf shapes); **C.** Flower and the linear-subulate apex with papillate; **D.** Fruit; **E.** Vittae number. Drawn by X.D. Ma.

**Figure 4.**
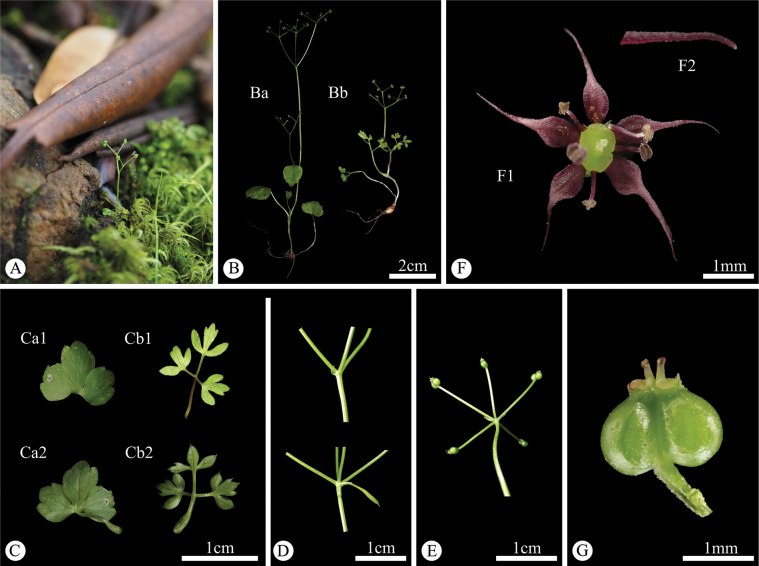
*Acronema handeloides* X.D.Ma & C.F.Song. **A.** Plant in natural habitat; **B.** Single individual; **C.** Basal leaves (**Ca1, Cb1.** Adaxial surface; **Ca2, Cb2.** Abaxial surface); **D.** Bracts and rays; **E.** Bracteoles and umbellules; **F.** Flower and petal apex (**F1.** Flower; **F2.** Linear-subulate apex with papillate); **G.** Fruit. Photographs by X.D. Ma.

##### Type.

China • Yunnan: Kunming City, Luquan County, Wumeng Township, Jiaozi Mountain, 26.0795°N, 102.8480°E, in the moss understory of the *Rhododendron* forest, 15 August 2025 (fl. & fr.), *X.D.Ma & Y.X MXD370* (holotype: NAS!; isotype: NAS!).

##### Description.

Annual to biennial herbs, glabrous, 1–10 cm tall. Roots spherical to ellipsoidal, 3–4 mm in diameter. Stems slender, with 1–2 lateral branches or unbranched. Basal leaves: petiole 8–30 mm, sheath narrow; leaf blade polymorphic, 1–1.5 cm long, 1.3–2 cm wide; when simple, blade broadly ovate, base truncate to slightly cordate, margin irregularly serrate, or trifid to tripartite, lobes obovate, apex trifid; when trifoliolate, leaflets short-petiolulate, apex trifid, lobes shallowly 1–3-lobed at apex; when 2-ternate, pinnately compound, ultimate segments obovate, apex trilobed. Cauline leaves similar to basal ones, occasionally simple and lanceolate. Peduncle 1.5–2.5 cm long; bracts 1 or absent, lanceolate; rays 2–5, 5–20 mm long, subequal; bracteoles 1–3, subulate; umbellules 2–5, 4–9 mm long, subequal. Calyx teeth subulate, conspicuous. Petals purplish red, ovate to obovate, ca. 1 mm long; apex linear-subulate, papillate, ca. 1 mm long. Fruit broadly ovoid, ca. 1 × 1.5 mm, base cordate, glabrous; primary ribs filiform, inconspicuous; vittae 1–2 in each furrow, 2 on the commissure.

##### Phenology.

Flowers are observed from July to August, and fruits from August to September.

##### Etymology.

The species epithet ‘handeloides’ is derived from the combination of “handel” (from *Acronema handelii*) and the suffix “-oides” (meaning “resembling”), indicating the morphological similarity of this new species to *A. handelii*. The Chinese name “拟中甸丝瓣芹” (nĭ zhōng diàn sī bàn qín) is a direct translation from the epithet, used to maintain nomenclatural consistency and avoid ambiguity.

##### Distribution and habitat.

**Figure 5.**
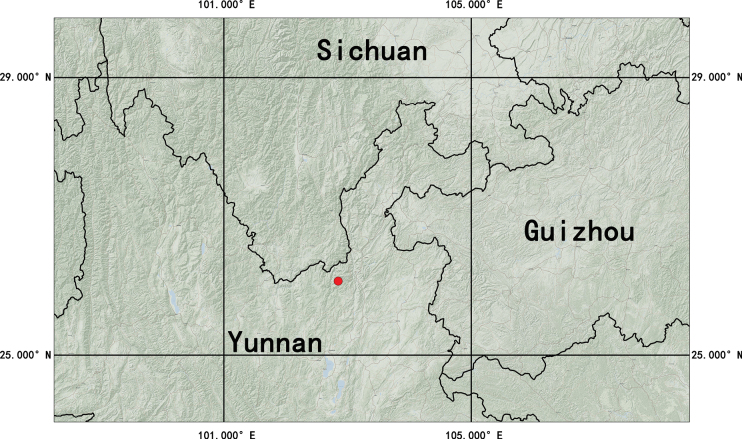
The distribution of *Acronema handeloides* (red circle).

This new species is currently only found in the moss understory of the Rhododendron forest at 3700 m on Jiaozi Mountain in Luquan County, Kunming, Yunnan (Fig. 5).

##### Additional material examined (paratype).

China • Yunnan: Kunming City, Luquan County, Wumeng Township, Jiaozi Mountain, 26.0795°N, 102.8480°E, on the moss understory of the *Rhododendron* forest, 31 August 2024 (fr.), *X.D.Ma & H.R.Zhuang MXD290* (NAS!).

##### Conservation status and discussion.

Following the description of *A. crassifolium* by Wang Huanchong in 2013, *A. handeloides* represents another new species of *Acronema* discovered on Jiaozi Mountain. Located near Kunming, Jiaozi Mountain is renowned as “the premier mountain of central Yunnan” and has been increasingly developed for tourism due to its scenic landscapes and winter snowfall (Chang 2019; Zhang et al. 2020). This development poses potential threats to understory habitats through intensified human activities. *Acronema* species typically occur in moist, moss-rich understories at high altitudes, representing a highly specialized ecological niche that is particularly vulnerable to disturbance in visited areas.

To date, *Acronema handeloides* is documented only from Jiaozi Mountain, with no prior collection records. Field observations indicate that the new species exhibits low fruit abortion rates in the wild, implying successful reproductive capacity under natural conditions. Despite this, available data on its full distribution and population trends remain incomplete. Therefore, in accordance with the IUCN Red List criteria (IUCN 2024), we recommend that the conservation status of *A. handeloides* be preliminarily assessed as Data Deficient (DD) to reflect current uncertainties.

## Supplementary Material

XML Treatment for Acronema handeloides

## ﻿Additional information

### Use of AI

No use of AI was reported.

### Author ORCIDs

Xu-Dong Ma https://orcid.org/0009-0006-6303-0006

Hui-Min Li https://orcid.org/0000-0001-6660-7620

Ying Xie https://orcid.org/0009-0004-1291-5432

Jun-Wen Zhu https://orcid.org/0009-0000-5554-445X

Jun Wen https://orcid.org/0000-0002-1926-6517

Wei Zhou https://orcid.org/0000-0003-1627-1529

Bao-Cheng Wu https://orcid.org/0009-0006-1719-3517

Chun-Feng Song https://orcid.org/0000-0003-2077-0486

### Data availability

All of the data that support the findings of this study are available in the main text.
